# Supplementary material for: A Combination of Machine Learning and PBPK Modeling Approach for Pharmacokinetics Prediction of Small Molecules in Humans
Source: Pharm Res. 2024 Jun 25;41(7):1369–79. doi: 10.1007/s11095-024-03725-y (PMC11534847; doi:10.1007/s11095-024-03725-y)
Supplement: Supplementary file 1 — (pdf 894 KB) [file 11095_2024_3725_MOESM1_ESM.pdf]

References

[1] Lombardo, F., Berellini, G., Obach, R.S.: Trend Analysis of a Database of Intravenous Pharmacokinetic Parameters in Humans for 1352 Drug Compounds. Drug Metab Dispos **46**(11), 1466–1477 (2018) https://doi.org/10.1124/dmd.118.082966

[2] Sohlenius-Sternbeck, A.-K., Afzelius, L., Prusis, P., Neelissen, J., Hoogstraate, J., Johansson, J., Floby, E., Bengtsson, A., Gissberg, O., Sternbeck, J., Petersson, C.: Evaluation of the human prediction of clearance from hepatocyte and microsome intrinsic clearance for 52 drug compounds. Xenobiotica **40**(9), 637–649 (2010) https://doi.org/10.3109/00498254.2010.500407 . Accessed 2023-06-14

[3] Mamada, H., Iwamoto, K., Nomura, Y., Uesawa, Y.: Predicting blood-to-plasma concentration ratios of drugs from chemical structures and volumes of distribution in humans. Molecular Diversity **25**(3), 1261–1270 (2021) https://doi.org/10.1007/s11030-021-10186-7 . Accessed 2023-09-26

[4] Murad, N., Pasikanti, K.K., Madej, B.D., Minnich, A., McComas, J.M., Crouch, S., Polli, J.W., Weber, A.D.: Predicting Volume of Distribution in Humans: Performance of In Silico Methods for a Large Set of Structurally Diverse Clinical Compounds. Drug Metabolism and Disposition **49**(2), 169–178 (2021) https://doi.org/10.1124/dmd.120.000202 . Accessed 2023-09-26

[5] PhamThe, H., Gonzlezlvarez, I., Bermejo, M., Garrigues, T., LeThiThu, H., CabreraPrez, M..: The Use of RuleBased and QSPR Approaches in ADME Profiling: A Case Study on Caco2 Permeability. Molecular Informatics **32**(5-6), 459–479 (2013) https://doi.org/10.1002/minf.201200166 . Accessed 2023-09-26

[6] OHagan, S., Kell, D.B.: The apparent permeabilities of Caco-2 cells to marketed drugs: magnitude, and independence from both biophysical properties and endogenite similarities. PeerJ **3**, 1405 (2015) https://doi.org/10.7717/peerj.1405 . Accessed 2023-06-12

[7] Bittermann, K., Goss, K.-U.: Predicting apparent passive permeability of Caco-2 and MDCK cell-monolayers: A mechanistic model. PLOS ONE **12**(12), 0190319 (2017) https://doi.org/10.1371/journal.pone.0190319 . Accessed 2023-09-26

[8] Hallifax, D., Foster, J.A., Houston, J.B.: Prediction of Human Metabolic Clearance from In Vitro Systems: Retrospective Analysis and Prospective View. Pharmaceutical Research **27**(10), 2150–2161 (2010) https://doi.org/10.1007/s11095-010-0218-3 . Accessed 2023-09-26

[9] Williamson, B., Harlfinger, S., McGinnity, D.F.: Evaluation of the Disconnect between Hepatocyte and Microsome Intrinsic Clearance and In Vitro In Vivo Extrapolation Performance. Drug Metabolism and Disposition **48**(11), 1137–1146 (2020) https://doi.org/10.1124/dmd.120.000131 . Accessed 2023-09-26

[10] Rawlins, M.D., Henderson, D.B., Hijab, A.R.: Pharmacokinetics of paracetamol (acetaminophen) after intravenous and oral administration. European Journal of Clinical Pharmacology **11**(4), 283–286 (1977) https://doi.org/10.1007/BF00607678 . Accessed 2023-06-12

[11] Adams, W.J., Bombardt, P.A., Brewer, J.E.: Normal-phase liquid chromatographic determination of alprazolam in human serum. Analytical Chemistry **56**(9), 1590–1594 (1984) https://doi.org/10.1021/ac00273a012 . Accessed 2023-06-12

[12] Jorgensen, A., Hansen, V.: Pharmacokinetics of amitriptyline infused intravenously in man. European Journal of Clinical Pharmacology **10**(5), 337–341 (1976) https://doi.org/10.1007/BF00565623 . Accessed 2023-06-12

[13] Kirch, W., Grg, K.G.: Clinical pharmacokinetics of atenolol A review. European Journal of Drug Metabolism and Pharmacokinetics **7**(2), 81–91 (1982) https://doi.org/10.1007/BF03188723 . Accessed 2023-06-12

[14] Ludden, T.M., Boyle, D.A., Gieseker, D., Kennedy, G.T., Crawford, M.H., Ludden, L.K., Clementi, W.A.: Absolute Bioavailability and Dose Proportionality of Betaxolol in Normal Healthy Subjects. Journal of Pharmaceutical Sciences **77**(9), 779–783 (1988) https://doi.org/10.1002/jps.2600770913 . Accessed 2023-06-12

[15] Weber, C., Schmitt, R., Birnboeck, H., Hopfgartner, G., Van Marle, S.P., Peeters, P.A.M., Jonkman, J.H.G., Jones, C.-R.: Pharmacokinetics and pharmacodynamics of the endothelin-receptor antagonist bosentan in healthy human subjects. Clinical Pharmacology & Therapeutics **60**(2), 124–137 (1996) https://doi.org/10.1016/S0009-9236(96)90127-7 . Accessed 2023-06-19

[16] Blanchard, J., Sawers, S.J.A.: Comparative pharmacokinetics of caffeine in young and elderly men. Journal of Pharmacokinetics and Biopharmaceutics **11**(2), 109–126 (1983) https://doi.org/10.1007/BF01061844 . Accessed 2023-06-12

[17] Yeung, P.K.-F., Hubbard, J.W., Korchinski, E.D., Midha, K.K.: Pharmacokinetics of chlorpromazine and key metabolites. European Journal of Clinical Pharmacology **45**(6), 563–569 (1993) https://doi.org/10.1007/BF00315316 . Accessed 2023-06-13

[18] Lebert, P.A., Mahon, W.A., MacLeod, S.M., Soldin, S.J., Fenje, P., Vandenberghe, H.M.: Ranitidine kinetics and dynamics: II. Intravenous dose studies and comparison with cimetidine. Clinical Pharmacology and Therapeutics **30**(4), 545–550 (1981) https://doi.org/10.1038/clpt.1981.201 . Accessed 2023-06-12

[19] Cheng, Y.F., Lundberg, T., Bondesson, U., Lindstrom, L., Gabrielsson, J.: Clinical pharmacokinetics of clozapine in chronic schizophrenic patients. European Journal of Clinical Pharmacology **34**(5), 445–449 (1988) https://doi.org/10.1007/BF01046700 . Accessed 2023-06-19

[20] Ciraulo, D.A., Barnhill, J.G., Jaffe, J.H.: Clinical pharmacokinetics of imipramine and desipramine in alcoholics and normal volunteers. Clinical Pharmacology and Therapeutics **43**(5), 509–518 (1988) https://doi.org/10.1038/clpt.1988.66 . Accessed 2023-06-19

[21] Spoorenberg, S.M.C., Deneer, V.H.M., Grutters, J.C., Pulles, A.E., Voorn, G.P.P., Rijkers, G.T., Bos, W.J.W., Van De Garde, E.M.W.: Pharmacokinetics of oral vs intravenous dexamethasone in patients hospitalized with community-acquired pneumonia: Pharmacokinetics of oral vs intravenous dexamethasone. British Journal of Clinical Pharmacology **78**(1), 78–83 (2014) https://doi.org/10.1111/bcp.12295 . Accessed 2023-06-19

[22] Andersson, T., Andrn, K., Cederberg, C., Edvardsson, G., Heggelund, A., Lundborg, R.: Effect of omeprazole and cimetidine on plasma diazepam levels. European Journal of Clinical Pharmacology **39**(1), 51–54 (1990) https://doi.org/10.1007/BF02657057 . Accessed 2023-06-12

[23] Willis, J.V., Kendall, M.J., Flinn, R.M., Thornhill, D.P., Welling, P.G.: The pharmacokinetics of diclofenac sodium following intravenous and oral administration. European Journal of Clinical Pharmacology **16**(6), 405–410 (1979) https://doi.org/10.1007/BF00568201 . Accessed 2023-06-19

[24] Hermann, P., Rodger, S.D., Remones, G., Thenot, J.P., London, D.R., Morselli, P.L.: Pharmacokinetics of diltiazem after intravenous and oral administration. European Journal of Clinical Pharmacology **24**(3), 349–352 (1983) https://doi.org/10.1007/BF00610053 . Accessed 2023-06-19

[25] Hammarlund, M.M., Paalzow, L.K., Odlind, B.: Pharmacokinetics of furosemide in man after intravenous and oral administration. Application of moment analysis. European Journal of Clinical Pharmacology **26**(2), 197–207 (1984) https://doi.org/10.1007/BF00630286 . Accessed 2023-06-12

[26] Martin, W., Koselowske, G., Tberich, H., Kerkmann, T., Mangold, B., Augustin, J.: Pharmacokinetics and absolute bioavailability of ibuprofen after oral administration of ibuprofen lysine in man. Biopharmaceutics & Drug Disposition **11**(3), 265–278 (1990) https://doi.org/10.1002/bdd.2510110311 . Accessed 2023-06-12

[27] Debruyne, D., De Ligny, B.H., Ryckelynck, J.-P., Albessard, F., Moulin, M.: Clinical Pharmacokinetics of Ketoprofen After Single Intravenous Administration as a Bolus or Infusion:. Clinical Pharmacokinetics **12**(3), 214–221 (1987) https://doi.org/10.2165/00003088-198712030-00003 . Accessed 2023-06-12

[28] Bennett, P.N., Aarons, L.J., Bending, M.R., Steiner, J.A., Rowland, M.: Pharmacokinetics of lidocaine and its deethylated metabolite: Dose and time dependency studies in man. Journal of Pharmacokinetics and Biopharmaceutics **10**(3), 265–281 (1982) https://doi.org/10.1007/BF01059261 . Accessed 2023-06-19

[29] Booker, B., Magee, M., Blum, R., Lates, C., Jusko, W.: Pharmacokinetic and pharmacodynamic interactions between diltiazem and methylprednisolone in healthy volunteers. Clinical Pharmacology & Therapeutics **72**(4), 370–382 (2002) https://doi.org/10.1067/mcp.2002.127944 . Accessed 2023-06-12

[30] Richard, J., Cardot, J.-M., Godbillon, J.: Inter- and intra-subject variability of metoprolol kinetics after intravenous administration. European Journal of Drug Metabolism and Pharmacokinetics **19**(2), 157–162 (1994) https://doi.org/10.1007/BF03188836 . Accessed 2023-06-13

[31] Smith, M.T., Eadie, M.J., Brophy, T.O.: The pharmacokinetics of midazolam in man. European Journal of Clinical Pharmacology **19**(4), 271–278 (1981) https://doi.org/10.1007/BF00562804 . Accessed 2023-06-13

[32] Cheng, H., Leff, J.A., Amin, R., Gertz, B.J., De Smet, M., Noonan, N., Rogers, J.D., Malbecq, W., Meisner, D., Somers, G.: Pharmacokinetics, bioavailability, and safety of montelukast sodium (MK-0476) in healthy males and females. Pharmaceutical Research **13**(3), 445–448 (1996) https://doi.org/10.1023/a:1016056912698

[33] Aitkenhead, A.R., Vater, M., Achila, K., Cooper, C.M.S., Smith, G.: PHARMACOKINETICS OF SINGLE-DOSE I.V.MORPHINE IN NORMAL VOLUNTEERS AND PATIENTS WITH END-STAGE RENAL FAILURE. British Journal of Anaesthesia **56**(8), 813–819 (1984) https://doi.org/10.1093/bja/56.8.813 . Accessed 2023-06-12

[34] Morrison, R.A., Singhvi, S.M., Creasey, W.A., Willard, D.A.: Dose proportionality of nadolol pharmacokinetics after intravenous administration to healthy subjects. European Journal of Clinical Pharmacology **33**(6), 625–628 (1988) https://doi.org/10.1007/BF00542499 . Accessed 2023-06-21

[35] Ngai, S.H., Berkowitz, B.A., Yang, J.C., Hempstead, J., Spector, S.: Pharmacokinetics of Naloxone in Rats and in Man. Anesthesiology **44**(5), 398–401 (1976) https://doi.org/10.1097/00000542-197605000-00008 . Accessed 2023-06-19

[36] Calvo, M., Lanao, J., Dominguezgil, A.: Bioavailability of rectally administered naproxen. International Journal of Pharmaceutics **38**(1-3), 117–122 (1987) https://doi.org/10.1016/0378-5173(87)90106-2 . Accessed 2023-06-19

[37] Kleinbloesem, C.H., Van Brummelen, P., Van De Linde, J.A., Voogd, P.J., Breimer, D.D.: Nifedipine: Kinetics and dynamics in healthy subjects. Clinical Pharmacology and Therapeutics **35**(6), 742–749 (1984) https://doi.org/10.1038/clpt.1984.105 . Accessed 2023-06-12

[38] Regrdh, C.G., Andersson, T., Lagerstrm, P.O., Lundborg, P., Sknberg, I.: The Pharmacokinetics of Omeprazole in HumansA Study of Single Intravenous and Oral Doses:. Therapeutic Drug Monitoring **12**(2), 163–172 (1990) https://doi.

org/10.1097/00007691-199003000-00010 . Accessed 2023-06-13

[39] Pritchard, J.F., Bryson, J.C., Kernodle, A.E., Benedetti, T.L., Powell, J.R.: Age and gender effects on ondansetron pharmacokinetics: Evaluation of healthy aged volunteers. Clinical Pharmacology and Therapeutics **51**(1), 51–55 (1992) https://doi.org/10.1038/clpt.1992.7 . Accessed 2023-06-12

[40] Meredith, P.A., Elliott, H.L., Kelman, A.W., Reid, J.L.: Application of pharmacokinetic–pharmacodynamic modelling for the comparison of quinazoline α –adrenoceptor agonists in normotensive volunteers. Journal of Cardiovascular Pharmacology **7**(3), 532–537 (1985) https://doi.org/10.1097/00005344-198505000-00019 . Accessed 2023-06-12

[41] Taegtmeyer, A.B., Haschke, M., Tchambaz, L., Buylaert, M., Tschpl, M., Beuers, U., Drewe, J., Krhenbhl, S.: A Study of the Relationship between Serum Bile Acids and Propranolol Pharmacokinetics and Pharmacodynamics in Patients with Liver Cirrhosis and in Healthy Controls. PLoS ONE **9**(6), 97885 (2014) https://doi.org/10.1371/journal.pone.0097885 . Accessed 2023-06-19

[42] Fremstad, D., Nilsen, O.G., Storstein, L., Amlie, J., Jacobsen, S.: Pharmacokinetics of quinidine related to plasma protein binding in man. European Journal of Clinical Pharmacology **15**(3), 187–192 (1979) https://doi.org/10.1007/BF00563104 . Accessed 2023-06-19

[43] Agency, E.M.: ASSESSMENT REPORT FOR REVATIO. https://www.ema.europa.eu/en/documents/variation-report/revatio

[44] Ogilvie, R.I.: Clinical Pharmacokinetics of Theophylline. Clinical Pharmacokinetics **3**(4), 267–293 (1978) https://doi.org/10.2165/00003088-197803040-00002 . Accessed 2023-06-12

[45] Smith, R.B., Kroboth, P.D., Varner, P.D.: Pharmacodynamics of Triazolam After Intravenous Administration. The Journal of Clinical Pharmacology **27**(12), 971–979 (1987) https://doi.org/10.1002/j.1552-4604.1987.tb05599.x . Accessed 2023-06-13

[46] Barbarash, R.A., Bauman, J.L., Fischer, J.H., Rondos, G.T., Batenhorst, R.L.: Near–total Reduction in Verapamil Bioavailability by Rifampin. Chest **94**(5), 954–959 (1988) https://doi.org/10.1378/chest.94.5.954 . Accessed 2023-06-12

[47] Gebbia, V., Puozzo, C.: Oral versus intravenous vinorelbine: clinical safety profile. Expert Opinion on Drug Safety **4**(5), 915–928 (2005) https://doi.org/10.1517/14740338.4.5.915 . Accessed 2023-06-12

Table 1 Drug-specific input parameters from in vitro experiments and prediction

Drug	LogP	pKa[1]	fu_{ob}[2]	fu_{pr}	BP[3]	$Caco2_{ob}$[4]	$Caco2_{pr}$	CLi[illegible]
Acetaminophen	0.91	9.46(a)	0.52	0.34	1.04	100	19.2	1[illegible]
Alprazolam	3.02	5.01(b)	0.29	0.17	0.78	25.5	51.7	1[illegible]
Amitriptyline	4.81	9.76(b)	0.07	0.07	0.86	54.7	23.1	1[illegible]
Atenolol	0.43	9.67(b)	0.94	0.58	1.12	1.60	1.00	1[illegible]
Betaxolol	2.54	9.67(b)	0.22	0.36	1.03	53.9	44.8	1[illegible]
Bosentan	4.94	5.8(a)	0.04	0.07	0.55	1.05	21.0	1[illegible]
Caffeine	-0.07	0(n)	0.64	0.46	1.00	40.1	33.6	0[illegible]
Chlorpromazine	5.40	9.40(b)	0.06	0.06	1.19	17.8	10.5	4[illegible]
Cimetidine	-0.11	6.91(~~a~~b)	0.78	0.76	0.97	4.48	3.00	8[illegible]
Clozapine	3.40	7.35(b)	0.06	0.04	0.81	30.7	31.5	1[illegible]
Desipramine	3.90	10.01(b)	0.16	0.14	0.93	29.5	14.5	1[illegible]
Dexamethasone	1.68	12.42(a)	0.198	0.25	0.93	15.0	16.5	8[illegible]
Diazepam	2.82	3.4(b)	0.02	0.05	0.81	41.0	50.1	1[illegible]
Diclofenac	4.26	4(a)	0.01	0.004	0.60	20.2	39.8	1[illegible]
Diltiazem	2.73	~~8.18(~~12.86(a)-8.18(b)	0.18	0.15	0.99	38.7	20.2	2[illegible]
Furosemide	1.75	4.25(a)	0.01	0.13	0.90	1.60	1.60	2[illegible]
Ibuprofen	3.97	5.53(a)	0.006	0.05	0.55	48.1	46.2	3[illegible]
Imipramine	4.28	9.2(b)	0.08	0.12	0.99	30.0	13.2	2[illegible]
Ketoprofen	3.10	4.45(a)	0.008	0.01	1.09	40.3	48.3	6[illegible]
Lidocaine	2.84	7.75(b)	0.33	0.28	0.74	18.5	21.5	1[illegible]
Methylprednisolone	1.80	0(n)	0.23	0.17	0.88	9.59	11.8	2[illegible]
Metoprolol	1.76	9.67(b)	0.88	0.61	1.15	34.2	12.3	3[illegible]
Midazolam	2.90	~~9.16~~6.19(b)	0.02	0.04	0.68	39.8	42.6	3[illegible]
Montelukast	8.49	4.40(a)-3.12(b)	0.002	0.003	0.55	76.0	5.20	9[illegible]
Morphine	0.89	8.21(b)	0.65	0.57	1.00	6.27	10.2	6[illegible]
Nadolol	0.81	9.76(b)	0.14	0.65	1.00	1.17	2.90	1[illegible]
Naloxone	1.67	~~7.84(~~10.07(a)-7.84(b)	0.54	0.49	1.43	21.5	21.9	1[illegible]
Naproxen	3.18	4.15(a)	0.002	0.01	0.51	52.8	41.9	1[illegible]
Nifedipine	2.20	0(n)	0.04	0.04	0.74	42.0	29.0	8[illegible]
Omeprazole	2.23	~~9.29(~~4.47(b)-9.29(a)	0.05	0.03	0.61	54.8	12.4	1[illegible]
Ondansetron	2.80	7.34(b)	0.27	0.24	0.79	110	107	2[illegible]
Prazosin	1.30	6.54(b)	0.06	0.15	0.70	7.72	10.8	6[illegible]
Propranolol	2.58	9.67(b)	0.13	0.17	0.89	45.0	31.8	1[illegible]
~~Ranitidine~~Quinidine	~~0.20~~ 2.51	~~7.8~~9.05(b)	~~0.95~~ 0.26	~~0.59~~0.09	~~1.00~~ 1.02	~~4.45~~21.0	~~0.70~~ 5.70	~~3~~[illegible]
~~Quinidine~~Ranitidine	~~2.51~~ 0.20	~~9.05~~7.8(b)	~~0.26~~ 0.95	~~0.09~~0.59	~~1.02~~ 1.00	~~21.0~~4.45	~~5.70~~ 0.70	~~2~~[illegible]
Sildenafil	1.87	11.14(a)-5.59(b)	0.04	0.06	0.81	55.0	43.9	5[illegible]
Theophylline	-0.02	8.81(a)	0.61	0.34	0.92	44.1	14.5	2[illegible]
Triazolam	3.63	4.26(b)	0.10	0.16	0.62	28.0	29.9	4[illegible]
Verapamil	3.79	8.92(b)	0.09	0.14	0.817	9.02	4.30	1[illegible]
Vinorelbine	4.80	8.66(b)	0.87	0.09	0.58	1.30	2.90	1[illegible]

[1] a=acid,b=base,n=neutral

[2] pr=predicted,ob=observed.Experimental Plasma protein fraction unbound from Lombardo et.al[1, 2].

[3] Experimental Blood:Plasma ratio obtained from previous studies[3, 4].

[4] Experimental Caco-2 cell permeability(10^{-6}cm/s) obtained from previous publications[5–7].

[5] Experimental Intrinsic hepatic clearance(ml/min/kg) from literature[2, 8, 9].

[6] Total plasma clearance(L/hr/kg) for drugs obtained from Lombardo et.al[1].

Table 2 Prediction results for in vitro and ML input parameters

	In Vitro[1]				ML[2]				
Drug	$RT_{1/2}$[3]	RAUC	RCL	RV	$RT_{1/2}$	RAUC	RCL	RV	Reference[4]
Acetaminophen	0.34	1.47	0.68	0.21	0.45	0.71	1.40	0.49	[10]
Alprazolam	2.05	0.24	4.10	8.50	1.72	0.36	2.77	4.92	[11]
Amitriptyline	0.43	1.24	0.81	0.26	1.01	0.17	5.87	3.49	[12]
Atenolol	2.36	0.58	1.74	1.92	0.64	0.40	2.53	0.88	[13]
Betaxolol	0.34	0.61	1.65	0.42	0.64	0.43	2.34	1.36	[14]
Bosentan	10.2	0.92	1.09	10.1	7.59	0.45	2.23	27.5	[15]
Caffeine	0.60	1.48	0.68	0.36	0.50	0.74	1.35	0.67	[16]
Chlorpromazine	2.43	5.95	0.17	0.34	4.64	1.78	0.56	2.59	[17]
Cimetidine	0.83	~~1.77 0.56 0.22~~ 0.81	0.81	0.97	1.23	~~0.60~~1.03	0.64	0.59	[18]
Clozapine	3.04	3.24	0.31	1.12	2.81	13.3	0.07	0.20	[19]
Desipramine	0.41	2.20	0.45	0.13	1.90	0.60	1.65	1.91	[20]
Dexamethasone	0.49	0.45	2.21	0.44	0.46	0.26	3.89	0.57	[21]
Diazepam	1.52	4.33	0.23	0.42	0.71	0.65	1.54	1.23	[22]
Diclofenac	2.88	5.76	0.17	0.34	2.77	3.81	0.26	0.52	[23]
Diltiazem	2.55	3.01	0.33	0.75	2.72	1.85	0.54	1.08	[24]
Furosemide	1.63	4.20	0.24	1.29	0.80	2.68	0.37	1.03	[25]
Ibuprofen	1.14	1.61	0.62	0.78	3.13	0.65	1.54	5.39	[26]
Imipramine	0.60	2.52	0.40	0.15	1.98	0.54	1.85	2.12	[20]
Ketoprofen	5.35	9.18	0.11	0.59	1.54	1.70	0.59	1.01	[27]
Lidocaine	2.26	2.94	0.34	0.55	3.67	1.12	0.89	2.68	[28]
Methylprednisolone	1.38	1.05	0.95	0.42	1.37	0.85	1.18	0.49	[29]
Metoprolol	3.13	5.91	0.17	0.52	1.96	2.63	0.38	0.62	[30]
Midazolam	2.99	0.74	1.36	1.77	9.85	1.37	0.73	5.51	[31]
Montelukast	7.73	0.05	20.6	58.3	0.85	0.29	3.48	0.93	[32]
Morphine	1.41	3.73	0.27	0.22	0.30	0.57	1.77	0.38	[33]
Nadolol	0.98	1.34	0.75	0.38	0.30	0.51	1.95	0.43	[34]
Naloxone	2.60	0.62	1.62	2.92	1.30	1.12	0.89	0.89	[35]
Naproxen	0.73	0.50	2.00	1.55	0.35	0.18	5.52	1.68	[36]
Nifedipine	0.81	1.95	0.51	0.36	1.36	0.60	1.67	0.59	[37]
Omeprazole	4.59	5.92	0.17	1.12	1.47	0.98	1.03	1.76	[38]
Ondansetron	1.44	0.88	1.13	1.45	7.01	1.71	0.58	4.90	[39]
Prazosin	1.20	2.35	0.43	0.49	0.68	0.98	1.02	0.45	[40]
Propranolol	1.78	5.39	0.19	0.39	1.49	1.68	0.59	0.93	[41]
Quinidine	0.82	0.55	1.83	1.03	1.05	0.50	2.00	0.87	[42]
Ranitidine	1.48	1.33	0.75	0.85	1.85	1.58	0.63	0.66	[18]
Sildenafil	1.13	1.33	0.75	0.32	1.33	0.65	1.55	0.45	[43]
Theophylline	0.22	0.43	2.31	0.50	0.21	0.22	4.51	0.92	[44]
Triazolam	3.46	0.24	4.25	8.38	7.45	0.39	2.54	16.6	[45]
Verapamil	1.32	1.51	0.66	0.47	2.00	0.92	1.09	1.58	[46]
Vinorelbine	0.99	0.13	7.72	6.15	1.14	1.00	1.00	1.03	[47]

[1]Drug-specific input parameters from in vitro experiments

[2]Drug-specific input parameters from ML prediction

[3]R=Ratio of Predicted/Observed

[3]References for drugs clinical pharmacokinetics

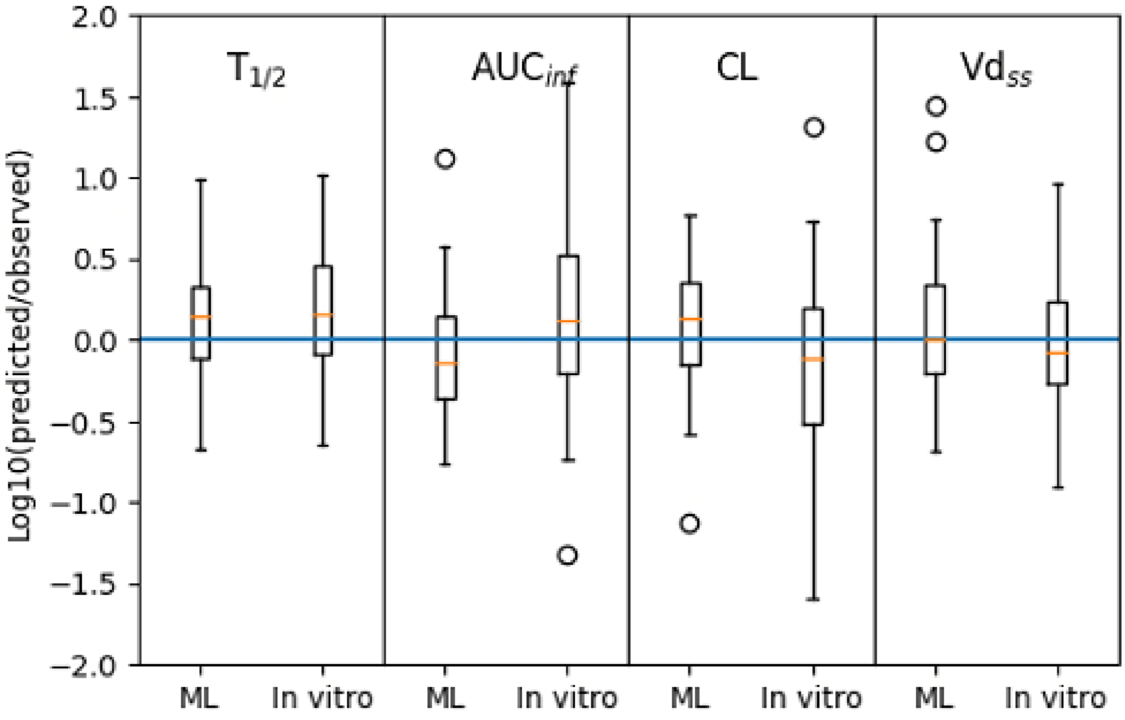

Fig. 1 Box plots present log ratios of predicted/observed PK parameter values. Positive values indicate overprediction, and negative values indicate underprediction. The blue line refers to the equal value between prediction and observation.

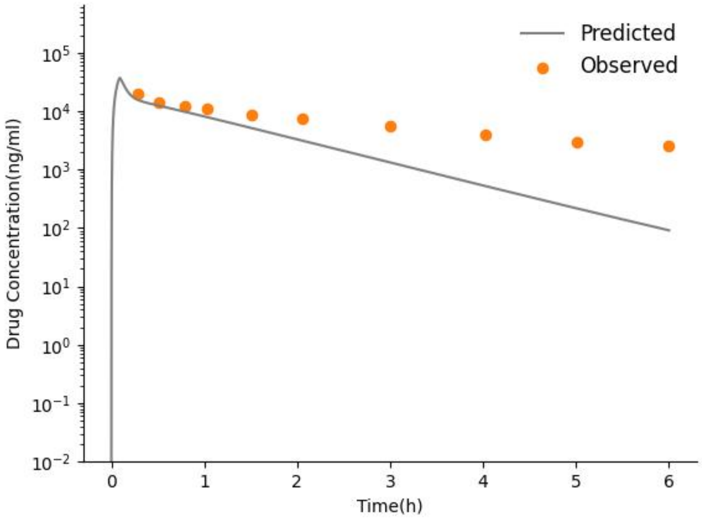

Fig. 2 Predicted vs. Observed Plot For Concentration-time Curve of Acetaminophen

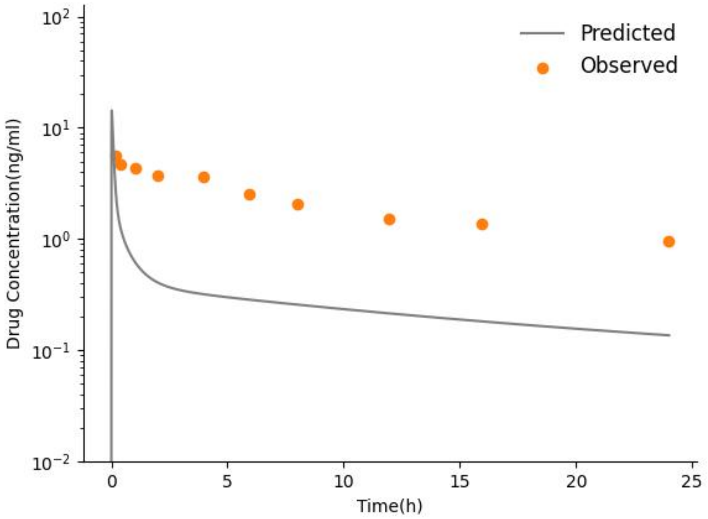

Fig. 3 Predicted vs. Observed Plot For Concentration-time Curve of Alprazolam

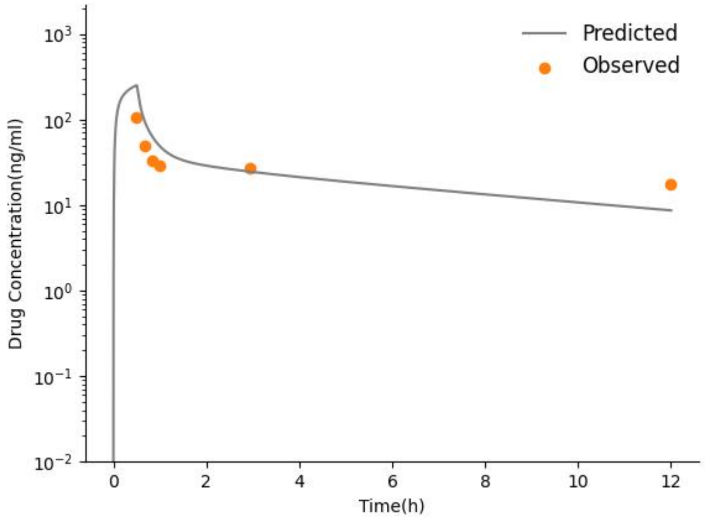

Fig. 4 Predicted vs. Observed Plot For Concentration-time Curve of Amitriptyline

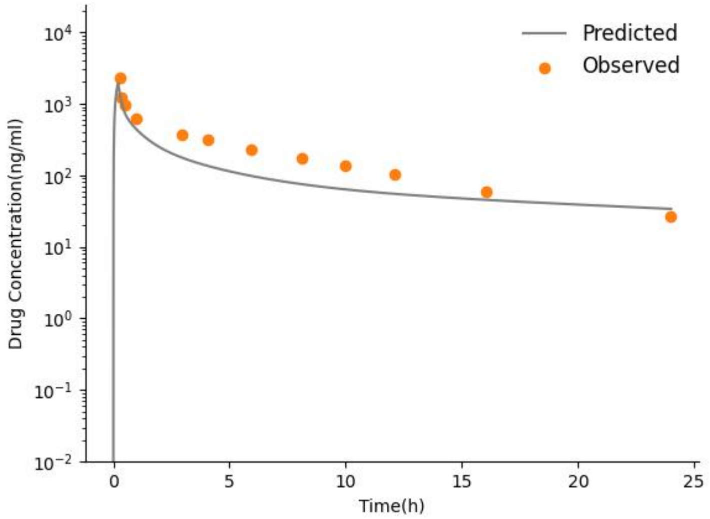

Fig. 5 Predicted vs. Observed Plot For Concentration-time Curve of Atenolol

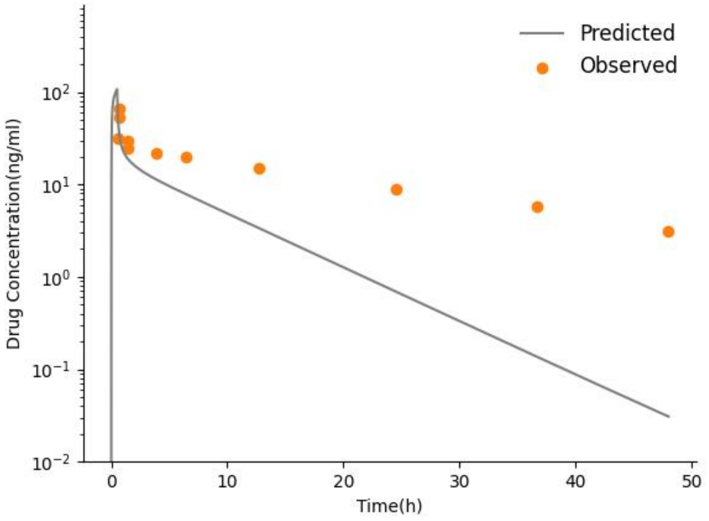

Fig. 6 Predicted vs. Observed Plot For Concentration-time Curve of Betaxolol

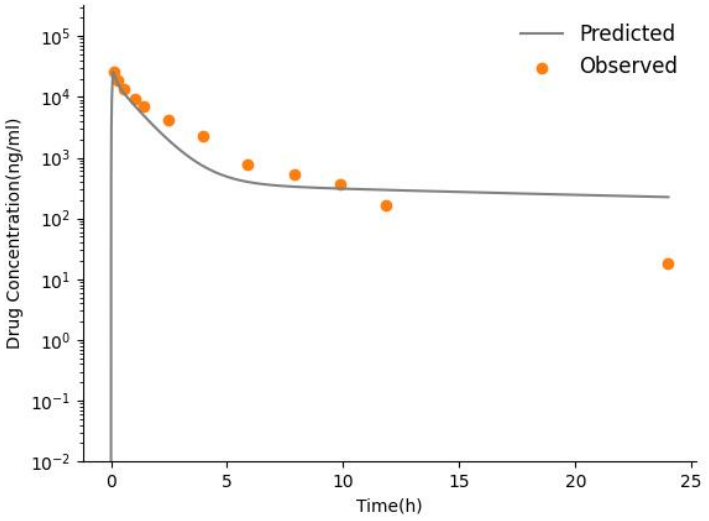

Fig. 7 Predicted vs. Observed Plot For Concentration-time Curve of Bosentan

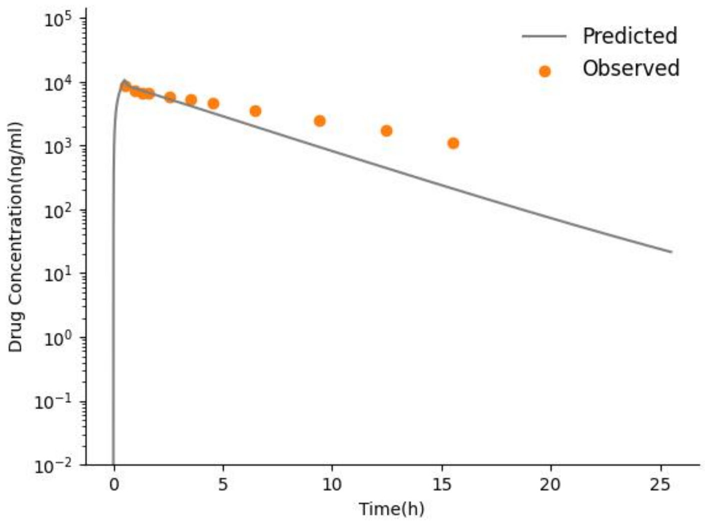

Fig. 8 Predicted vs. Observed Plot For Concentration-time Curve of Caffeine

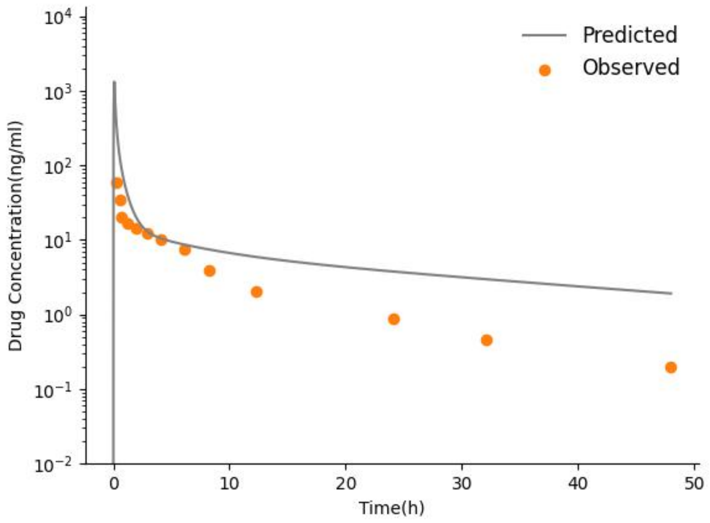

Fig. 9 Predicted vs. Observed Plot For Concentration-time Curve of Chlorpromazine

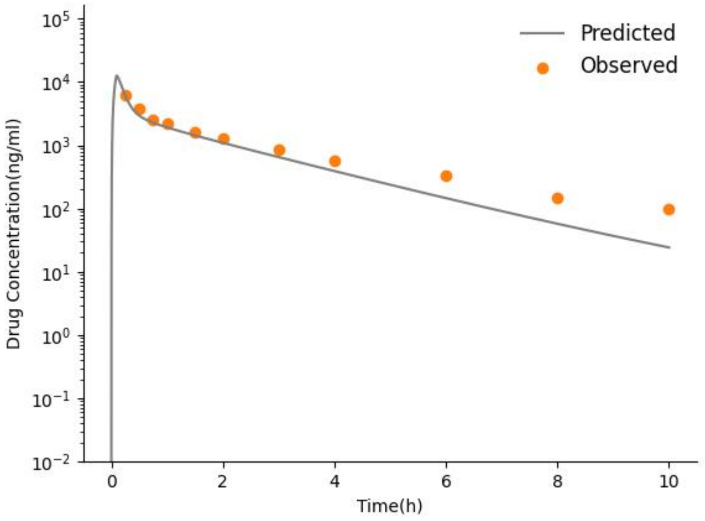

Fig. 10 Predicted vs. Observed Plot For Concentration-time Curve of Cimetidine

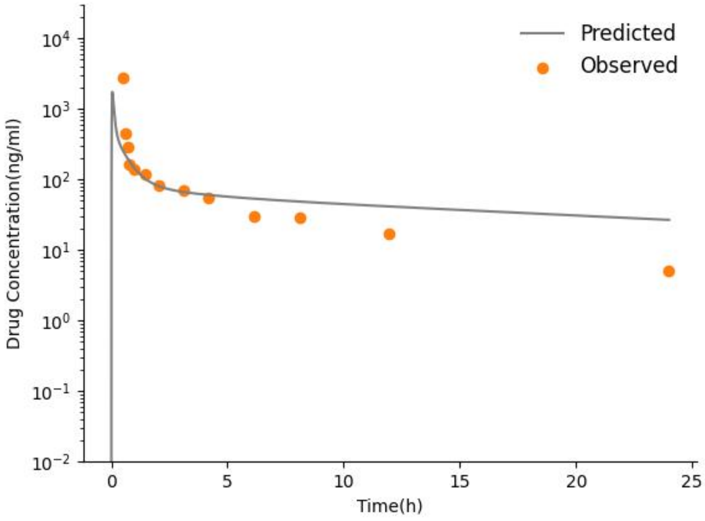

Fig. 11 Predicted vs. Observed Plot For Concentration-time Curve of Clozapine

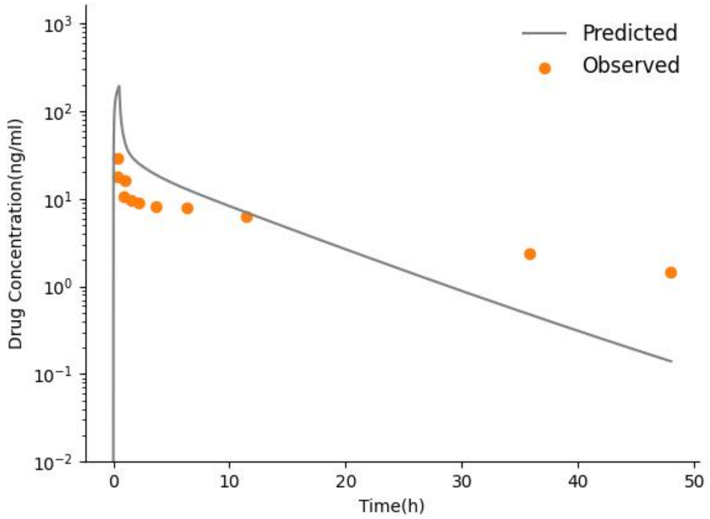

Fig. 12 Predicted vs. Observed Plot For Concentration-time Curve of Desipramine

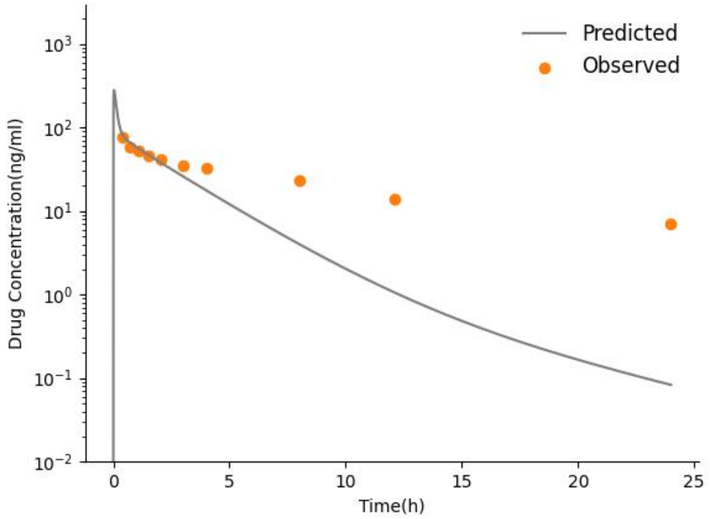

Fig. 13 Predicted vs. Observed Plot For Concentration-time Curve of Dexamethasone

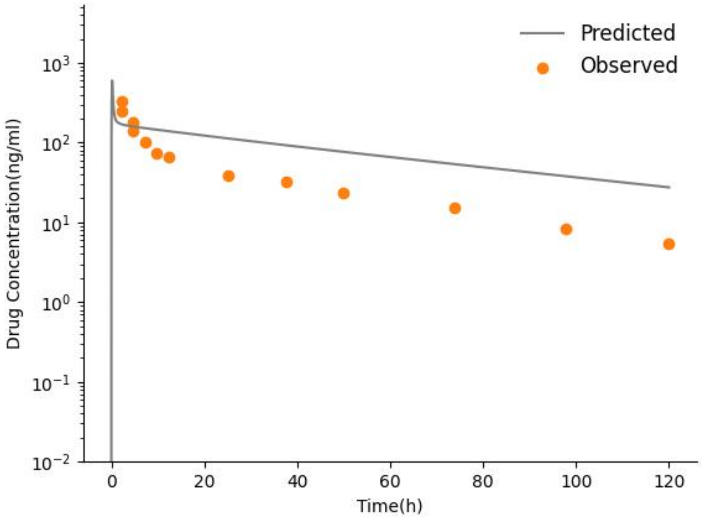

Fig. 14 Predicted vs. Observed Plot For Concentration-time Curve of Diazepam

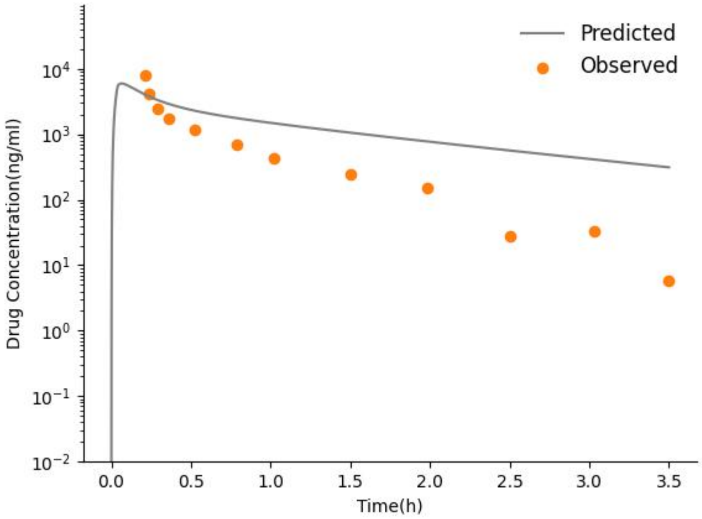

Fig. 15 Predicted vs. Observed Plot For Concentration-time Curve of Diclofenac

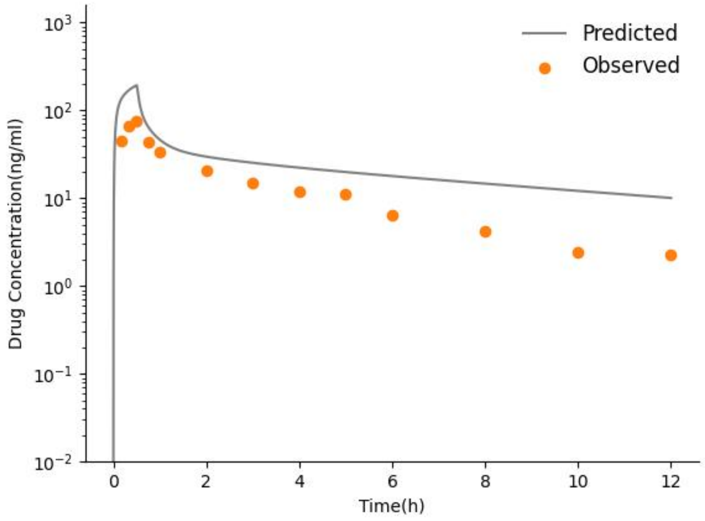

Fig. 16 Predicted vs. Observed Plot For Concentration-time Curve of Diltiazem

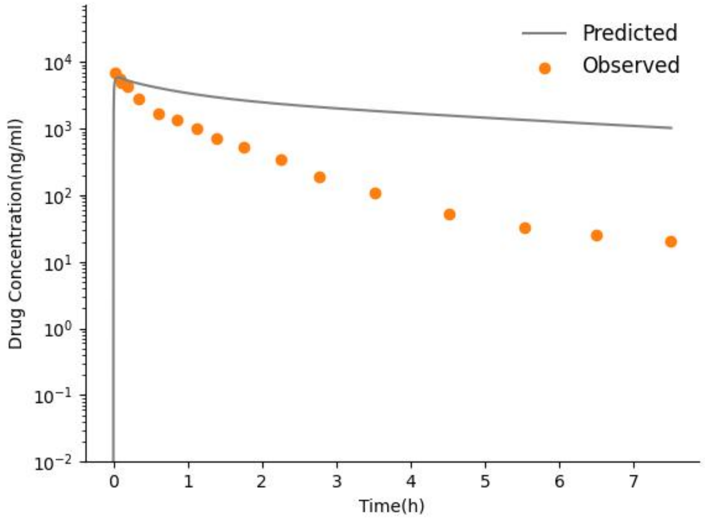

Fig. 17 Predicted vs. Observed Plot For Concentration-time Curve of Furosemide

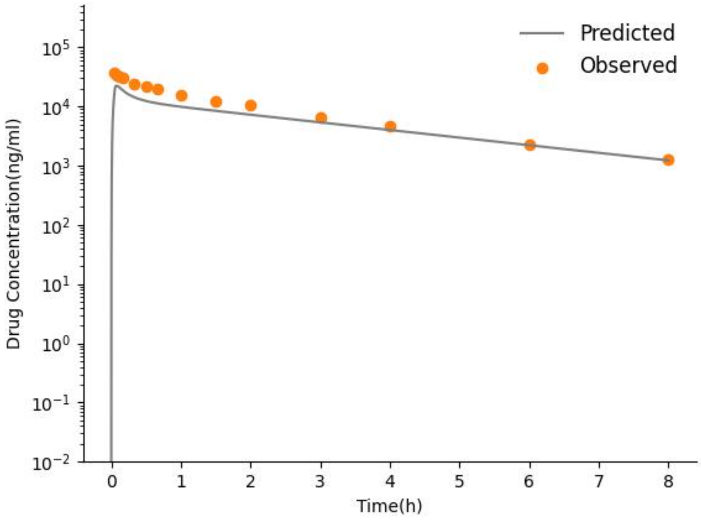

Fig. 18 Predicted vs. Observed Plot For Concentration-time Curve of Ibuprofen

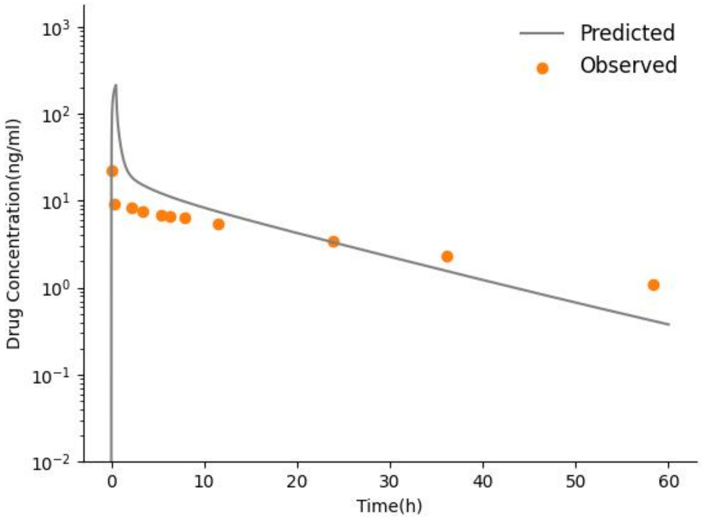

Fig. 19 Predicted vs. Observed Plot For Concentration-time Curve of Imipramine

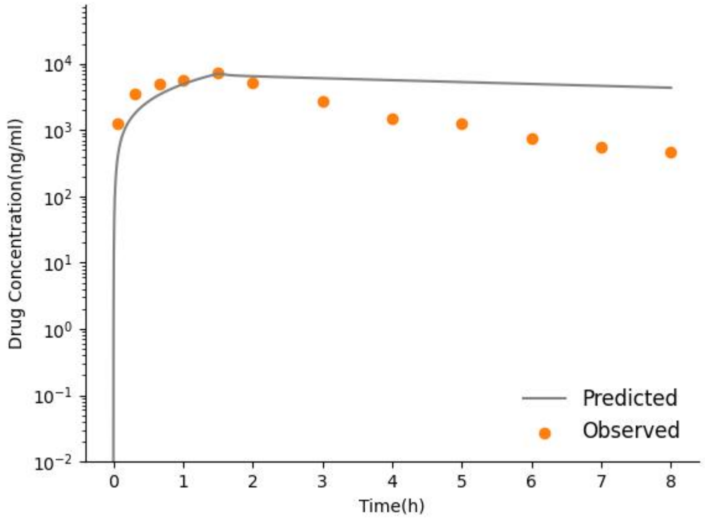

Fig. 20 Predicted vs. Observed Plot For Concentration-time Curve of Ketoprofen

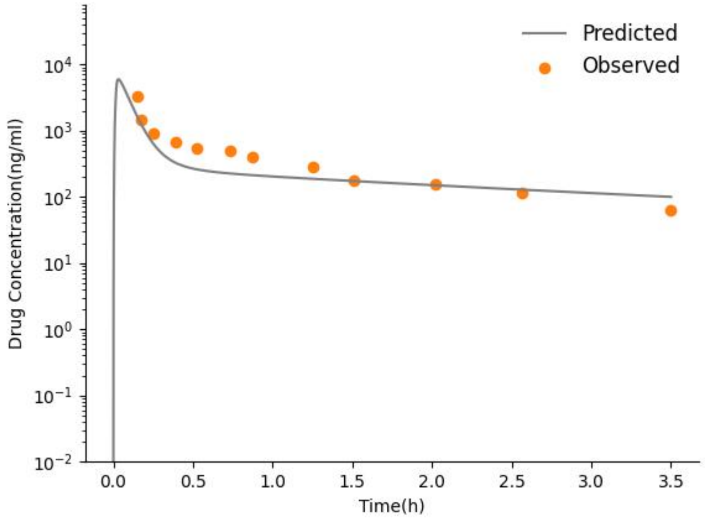

Fig. 21 Predicted vs. Observed Plot For Concentration-time Curve of Lidocaine

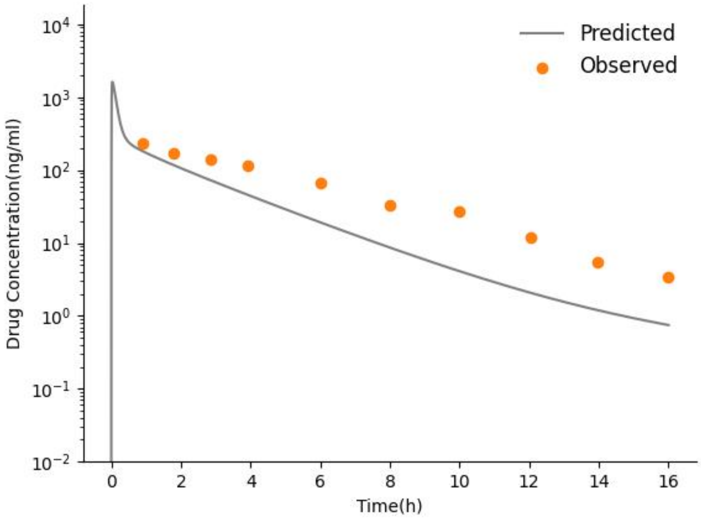

Fig. 22 Predicted vs. Observed Plot For Concentration-time Curve of Methylprednisolone

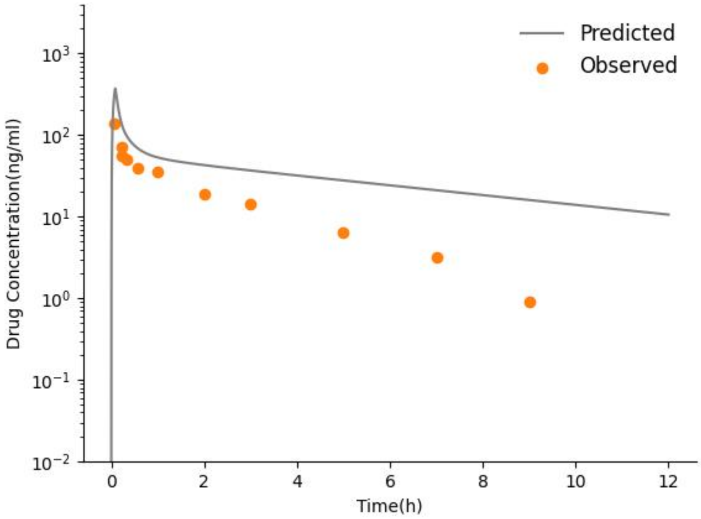

Fig. 23 Predicted vs. Observed Plot For Concentration-time Curve of Metoprolol

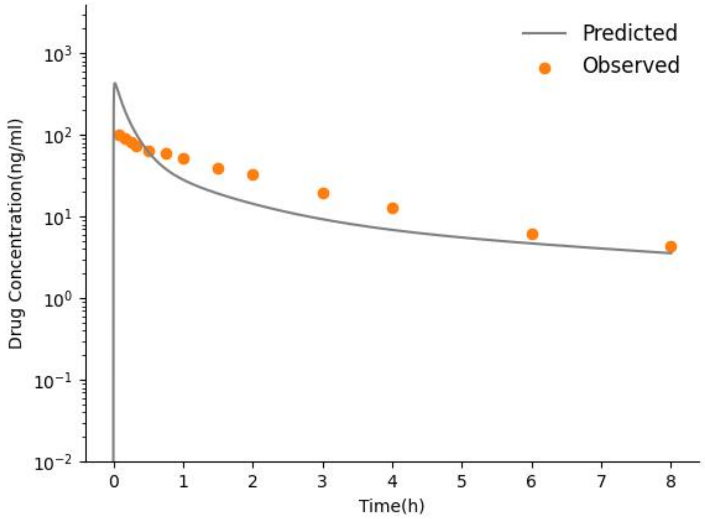

Fig. 24 Predicted vs. Observed Plot For Concentration-time Curve of Midazolam

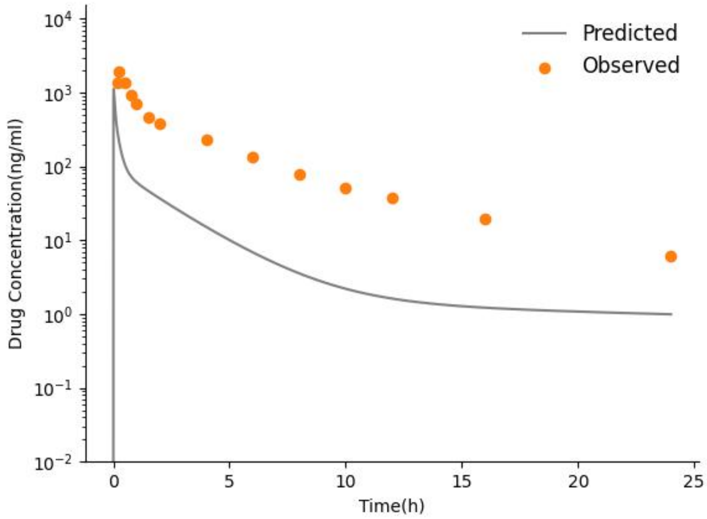

Fig. 25 Predicted vs. Observed Plot For Concentration-time Curve of Montelukast

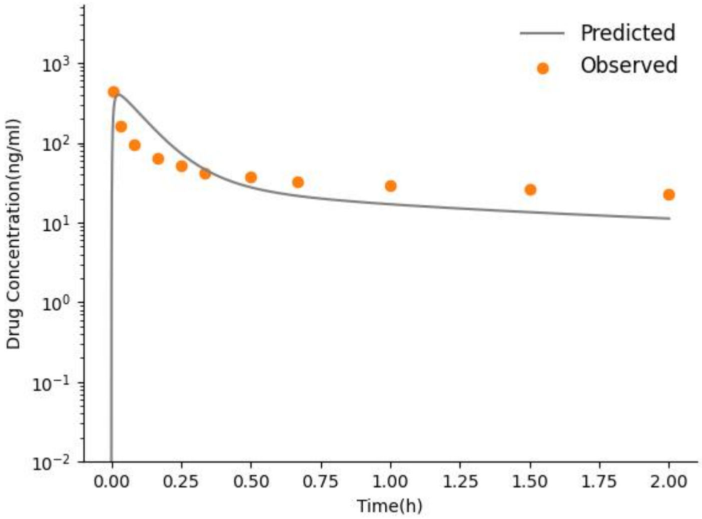

Fig. 26 Predicted vs. Observed Plot For Concentration-time Curve of Morphine

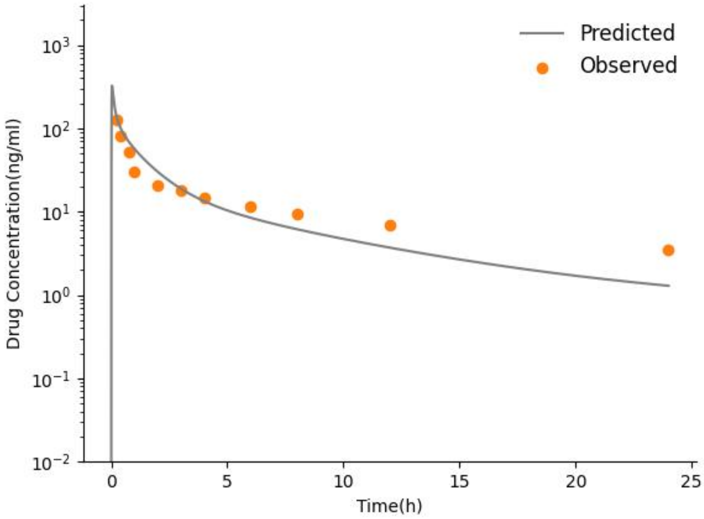

Fig. 27 Predicted vs. Observed Plot For Concentration-time Curve of Nadolol

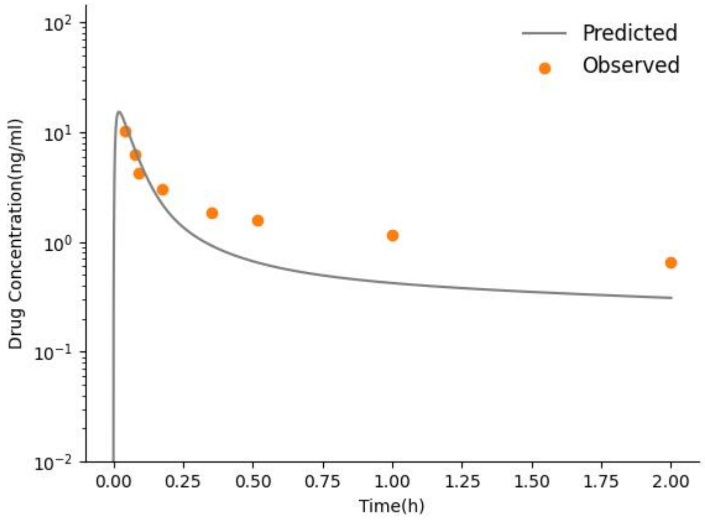

Fig. 28 Predicted vs. Observed Plot For Concentration-time Curve of Naloxone

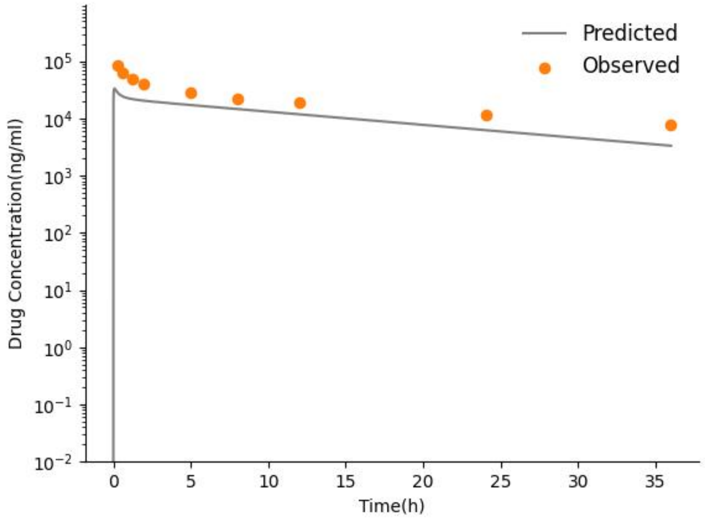

Fig. 29 Predicted vs. Observed Plot For Concentration-time Curve of Naproxen

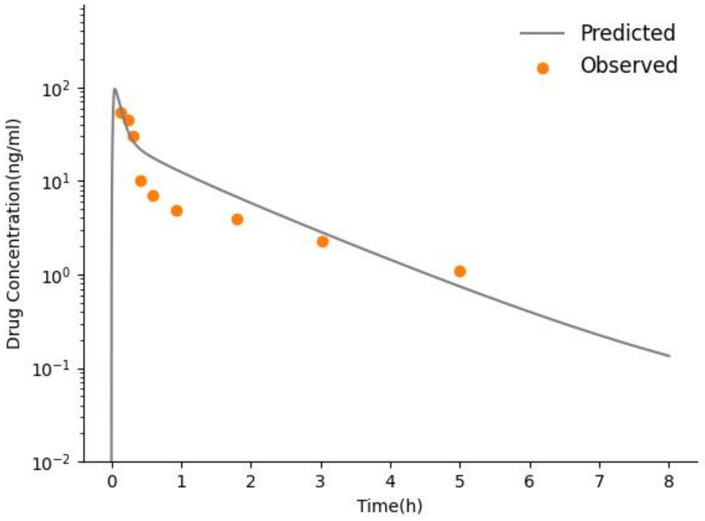

Fig. 30 Predicted vs. Observed Plot For Concentration-time Curve of Nifedipine

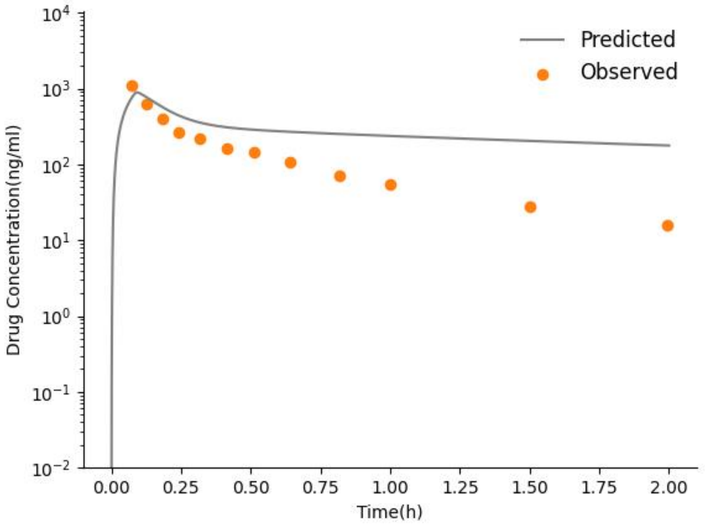

Fig. 31 Predicted vs. Observed Plot For Concentration-time Curve of Omeprazole

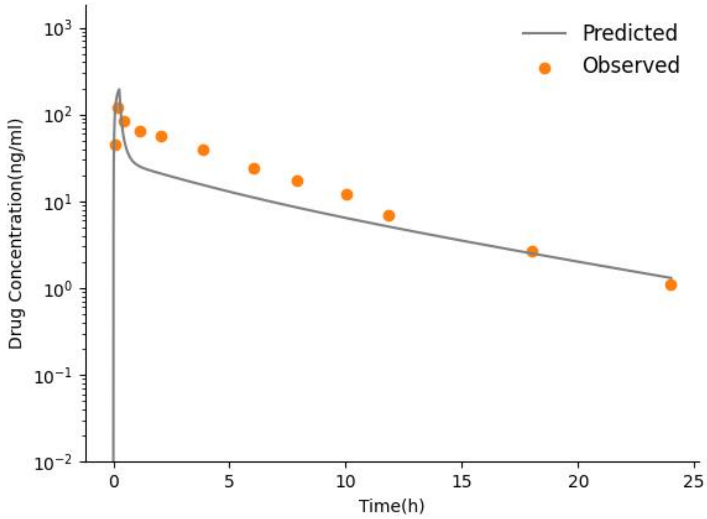

Fig. 32 Predicted vs. Observed Plot For Concentration-time Curve of Ondansetron

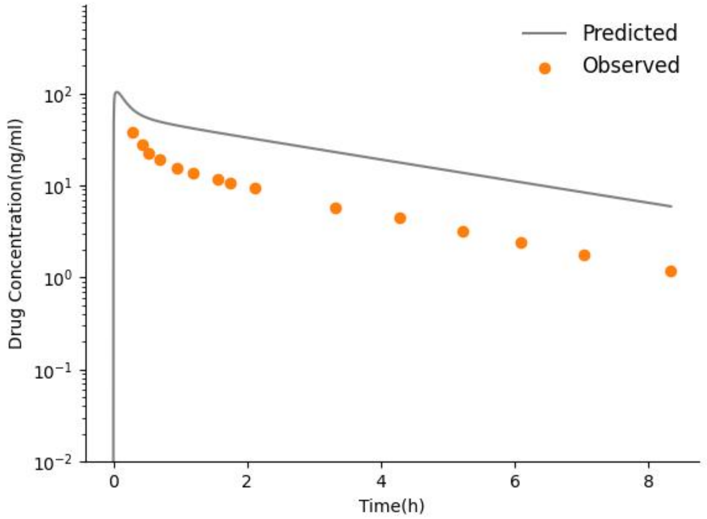

Fig. 33 Predicted vs. Observed Plot For Concentration-time Curve of Prazosin

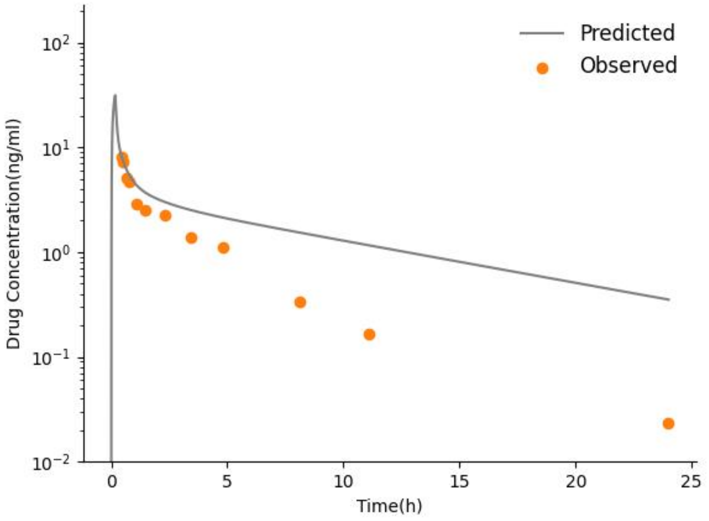

Fig. 34 Predicted vs. Observed Plot For Concentration-time Curve of Propranolol

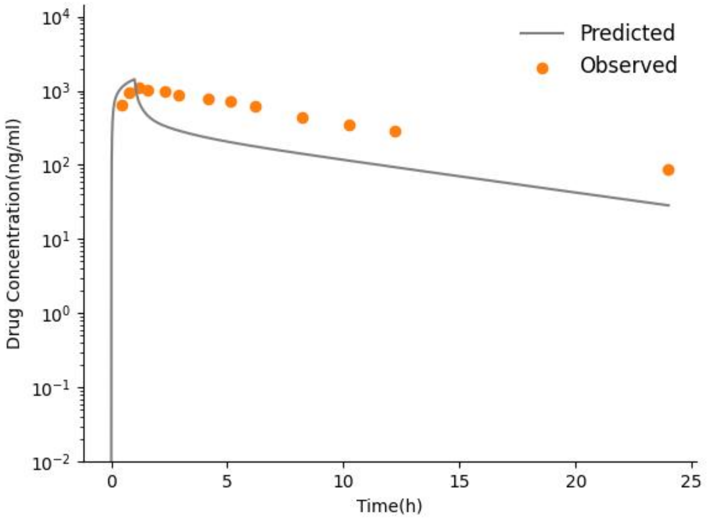

Fig. 35 Predicted vs. Observed Plot For Concentration-time Curve of Quinidine

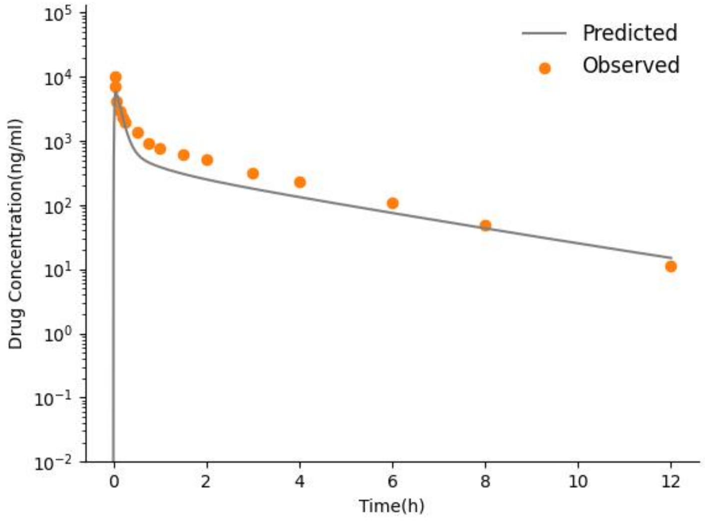

Fig. 36 Predicted vs. Observed Plot For Concentration-time Curve of Ranitidine

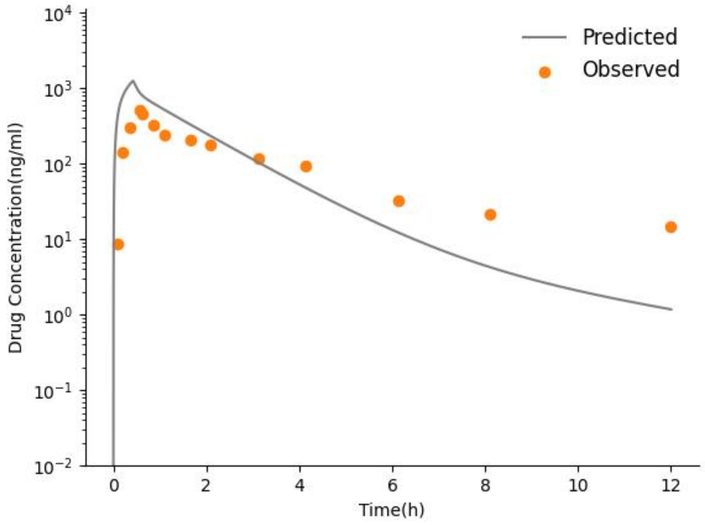

Fig. 37 Predicted vs. Observed Plot For Concentration-time Curve of Sildenafil

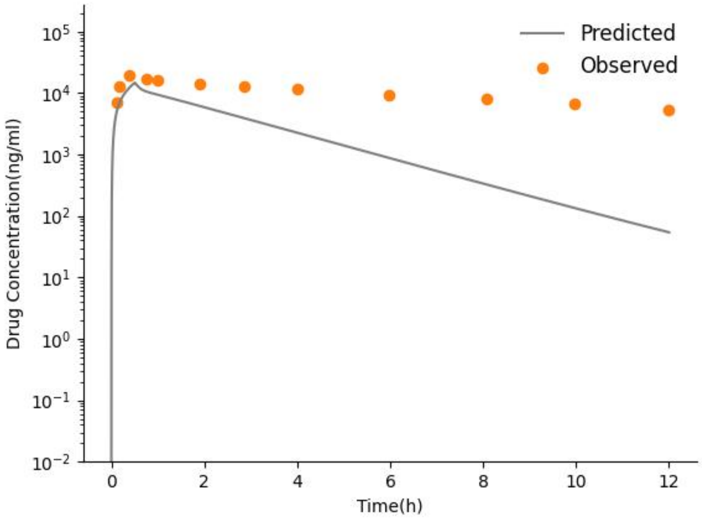

Fig. 38 Predicted vs. Observed Plot For Concentration-time Curve of Theophylline

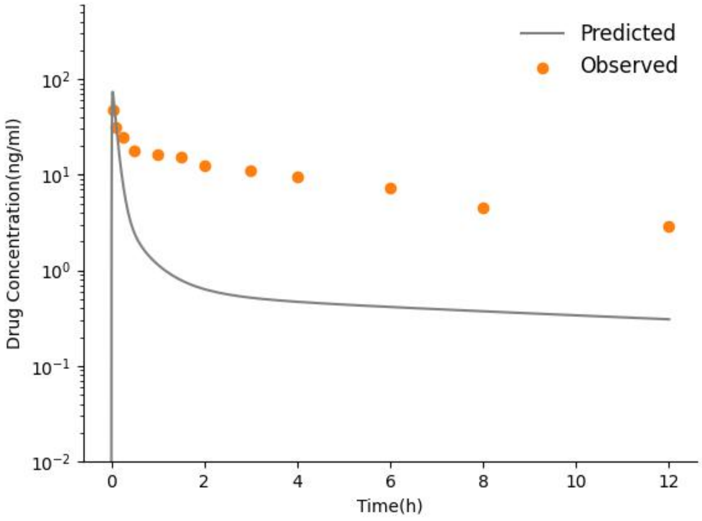

Fig. 39 Predicted vs. Observed Plot For Concentration-time Curve of Triazolam

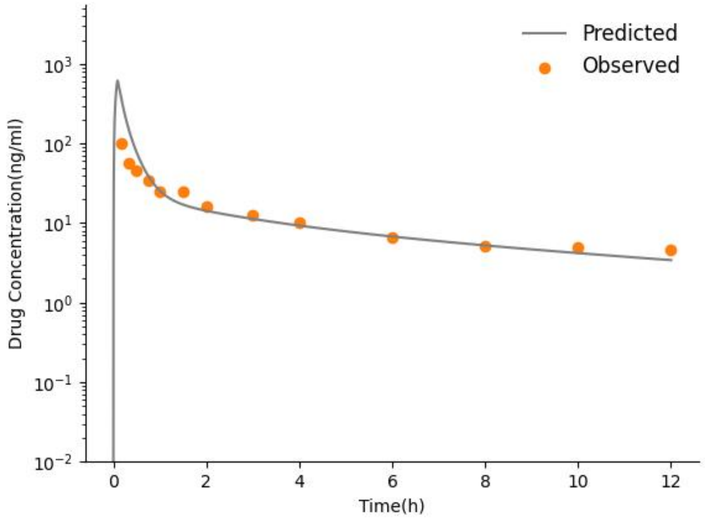

Fig. 40 Predicted vs. Observed Plot For Concentration-time Curve of Verapamil

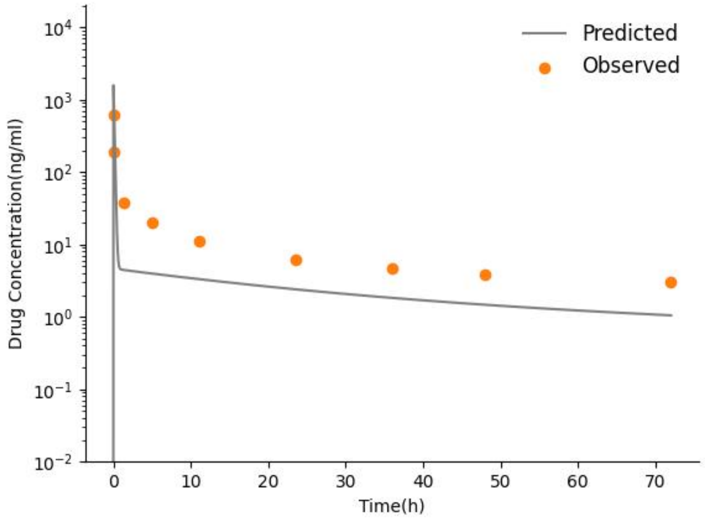

Fig. 41 Predicted vs. Observed Plot For Concentration-time Curve of Vinorelbine
